# Population regulation in *Gyrodactylus salaris* – Atlantic salmon (*Salmo salar* L.) interactions: testing the paradigm

**DOI:** 10.1186/s13071-015-0981-4

**Published:** 2015-07-25

**Authors:** Raúl Ramírez, Tor A. Bakke, Philip D. Harris

**Affiliations:** Natural History Museum, Department of Research and Collections, University of Oslo, P.O. Box 1172, NO-0318 Oslo, Norway

**Keywords:** Density-dependence, Time-dependence, Host-parasite dynamics, Transmission biology, Local adaptation

## Abstract

**Background:**

*Gyrodactylus salaris* is a directly transmitted ectoparasite that reproduces *in situ* on its fish host. Wild Norwegian (East Atlantic) salmon stocks are thought to be especially susceptible to the parasite due to lack of co-adaptation, contrary to Baltic salmon stocks. This study i) identifies whether time- and density-dependent mechanisms in gyrodactylid population growth exist in *G. salaris*-Atlantic salmon interactions and ii) based on differences between Norwegian and Baltic stocks, determines whether the ‘Atlantic susceptible, Baltic resistant’ paradigm holds as an example of local adaptation.

**Methods:**

A total of 18 datasets of *G. salaris* population growth on individually isolated Atlantic salmon (12 different stocks) infected with three parasite strains were re-analysed using a Bayesian approach. Datasets included over 2000 observations of 388 individual fish.

**Results:**

The best fitting model of population growth was time-limited; parasite population growth rate declined consistently from the beginning of infection. We found no evidence of exponential population growth in any dataset. In some stocks, a density dependence in the size of the initial inoculum limited the maximum rate of parasite population growth. There is no evidence to support the hypothesis that all Norwegian and Scottish Atlantic salmon stocks are equally susceptible to *G. salaris*, while Baltic stocks control and limit infections due to co-evolution. Northern and Western Norwegian as well as the Scottish Shin stocks, support higher initial parasite population growth rates than Baltic, South-eastern Norwegian, or the Scottish Conon stocks, and several Norwegian stocks tested (Akerselva, Altaelva, Lierelva, Numedalslågen), and the Scottish stocks (i.e. Conon, Shin), were able to limit infections after 40–50 days. No significant differences in performance of the three parasite strains (Batnfjordselva, Figga, and Lierelva), or the two parasite mitochondrial haplotypes (A and F) were observed.

**Conclusions:**

Our study shows a spectrum of growth rates, with some fish of the South-eastern Norwegian stocks sustaining parasite population growth rates overlapping those seen on Baltic Neva and Indalsälv stocks. This observation is inconsistent with the ‘Baltic-resistant, Atlantic-susceptible’ hypothesis, but suggests heterogeneity, perhaps linked to other host resistance genes driven by selection for local disease syndromes.

**Electronic supplementary material:**

The online version of this article (doi:10.1186/s13071-015-0981-4) contains supplementary material, which is available to authorized users.

## Background

The monogenean *Gyrodactylus salaris* Malmberg, 1957 has been an important pathogen of juvenile freshwater stages of Atlantic salmon, *Salmo salar* L. in Norway since the 1970s, when it is thought to have been introduced from the Baltic region [1]. An early conclusion was that, following the introduction of this parasite, Norwegian (Eastern Atlantic) salmon stocks were especially susceptible due to a lack of co-adaptative evolutionary experience of *G. salaris*, contrary to Baltic salmon stocks [2]. This hypothesis has been repeated, with numerous studies comparing parasite performance on different salmon stocks and other salmonids, generating one of the largest available data sets using a common garden experimental approach to examine host specificity of a metazoan parasite infecting vertebrates. Studies with other gyrodactylid-host systems have shown that parasite infra-populations grow rapidly to a peak, followed by decline and eventual elimination [3–14], a pattern interpreted as exponential increase before induction of immunity after a short period of recognition and activation [15–19]. The continuous growth of *G. salaris* on susceptible Norwegian salmon was therefore interpreted as evidence of impaired immunity [20], despite earlier suggestions that even on these fish, parasite population growth rates could decline throughout infection [21]. The resulting ‘Atlantic susceptible, Baltic resistant’ paradigm has become firmly entrenched. Resistance to *G. salaris* has been described as the ‘most convincing example of adaptive variation leading to local adaptation in Atlantic salmon’ [22], while Peeler *et al*. [23] consider this parasite to be the greatest threat posed to susceptible Eastern Atlantic salmon stocks in the UK. At a practical level, the paradigm has driven the rotenone eradication campaign in Norway, which has been applied to both infected rivers and large lake systems, and the costly surveillance and public education programmes implemented in many other northern European nations such as Scotland [24].

An assumption of all previous experimental work has been that differences between fish and fish stocks have an underlying genetic cause [2, 5, 7, 11, 25–27] and that the common garden methodology with isolated hosts can detect this [2]. Indeed, this assumption of a genetic basis to susceptibility/resistance is fundamental to the concept of local adaptation to parasites [28]. Studies with gyrodactylids have, however, all used a frequentist (mean variance) statistical framework in which it is difficult to identify phenotypic differences in host susceptibility between individuals, which may correlate with genotype. In particular, the auto-correlative nature of gyrodactylid population growth, and small differences between hosts in the timing of parasite population growth have major effects on the outcome of infections [29], which cannot be captured by frequentist methodologies. At the same time, a key issue with the common garden approach is the nature of experimental replication [30]. In the case of *Gyrodactylus*, most published studies consist of single studies with fish infected simultaneously in a single laboratory. There are no published studies replicating susceptibility trials in space and time, which could allow realistic estimation of genuine genetic stock effects as opposed to experimental, environmental replicate effects. In this paper we therefore set out to re-evaluate the corpus of data on common garden experiments, some unpublished, using *G. salaris* on different Atlantic salmon stocks performed since the late 1980s, carried out by researchers from the Natural History Museum Oslo (NHMO). We applied a Bayesian statistical methodology to avoid the pitfalls of the frequentist analytical approach used in the original papers, and we included datasets featuring the same salmon stock infected at different times under different conditions to allow appraisal of stock replicability. We were especially interested in establishing (a) whether there is any time-dependent limitation of gyrodactylid population growth consistent with an immune response; and (b), whether any density-dependent regulation of parasite population growth can be detected. In particular, we focused on the differences between salmon stocks from Scotland and Fennoscandia to determine whether the ‘Atlantic susceptible, Baltic resistant’ paradigm of resistance to *G. salaris* can be sustained as the ‘most convincing example of adaptive variation leading to local adaptation in Atlantic salmon’[22].

## Methods

### Infection experiments and parasite strains

All experiments were conducted between 1989 and 2013 (Table 1). Most have been published individually, but several are unpublished. The majority were carried out at NHMO, the remainder, with imported salmon stocks and their controls, at the VESO Vikan facility (Namsos city, Nord-Trøndelag County, Norway). Experiments [approved by the Norwegian Animal Research Authority (Forsøksdyrutvalget, FDU), licence ID Saksnr. 2012/279509] utilised primarily the Southeast Norwegian Lierelva strain (parasite strain nomenclature based on river of origin) of *G. salaris* (mitochondrial haplotype F [31]), or for experiments conducted at Vikan VESO, the central Norwegian Figga strain (haplotype A). Two datasets (Batnfjordselva and Lierelva stock experiments conducted at NHMO) utilised a second haplotype A parasite strain, from the Southwestern Norwegian Batnfjordselva River. Parasites were collected by electrofishing of heavily infected salmon parr and maintained in the laboratory for periods ranging from several weeks to a few months before experiments began. All experiments were identical except for details of the infection process, and were performed in plastic aquaria (100 × 100 × 20 cm water level), within which fish were individually isolated in floating enclosures (20 × 10 × 10 cm water level) with wire mesh bottoms to ensure a common garden environment (see [27]). Water temperature was maintained at 11 ± 0.5 °C, with continous dim illumination. Fish were fed ad libitum on pelleted food (EWOS) prior to experiments, but were fasted during the period of observation.

**Table 1:** Overview of the experimental data-sets analysed

**Data-set**	Date of experiments	Fish stock and replicate	Parasite strain and haplotype [ ]	Length of experiment (days)	Number of fish used	Initial parasite load	Max parasite load	References
A	1989	Loneelva single worm	Lierelva [F]	28	10	1	69	2
Bi	1993	Batnfjordselva	Batnfjordselva [A]	36	23	21-308	2500	Unpublished
Bii	1993	Lierelva	Batnfjordselva [A]	36	24	30-422	1400	Unpublished
C	1993	Imsa	Lierelva [F]	35	18	16-221	1100	Unpublished
D	1994	Altaelva	Lierelva [F]	42	24	23-111	753	20
Ei	1998	Akerselva	Lierelva [F]	42	21	22-176	2000	Unpublished
Eii	1998	Akerselva	Lierelva [F]	35	40	31-170	1000	Unpublished
Eiii	1998	Akerselva	Lierelva [F]	35	39	4-44	500	Unpublished
F	1991	Conon	Figga [A]	49	24	31-116	4000	19
G	1991	Shin	Figga [A]	49	24	33-236	1400	19
H	2013	Numedalslågen	Lierelva [F]	84	16	5	772	10
I	1992	Lierelva	Figga [A]	49	24	29-184	2000	19
J	1992	Indalsälv	Figga [A]	35	24	77-161	1600	21
K	1994	Neva	Lierelva [F]	51	38	12-180	260	20
L	1989	Neva single worm	Lierelva [F]	28	7	1	14	2
M	1995	Neva	Lierelva [F]	51	12	2-10	150	Unpublished
N	1990	Namsen	Lierelva [F]	21	7	9-38	266	Unpublished
O	1995	Altaelva	Lierelva [F]	51	12	3-12	400	Unpublished

The experimental details are provided for each data-set

Three approaches to infection were followed. In the earliest experiments fish were infected by allowing them to swim with infected donor fish before separation into the individual enclosures, after which fish were anaesthetised once per week using 0.04 % chlorbutanol and all parasites were counted. This approach resulted in considerable variation in the initial parasite inoculum, and so in later experiments fish were allowed to swim for 24 hours amongst heavily infected fins clipped from previously killed donors. With experience, manipulation of the number of parasites added resulted in relatively consistent initial inocula. Finally, for experiments with specified starting inocula (e.g. single worm infections), individual worms were manipulated from a donor onto insect pins and then transferred onto a fin of an anaesthetised experimental host. When the appropriate number of worms had been added, the experimental fish was returned to the individual enclosure, and was checked after 24 hours to ensure that the worm or worms had attached and not been lost. Infections were continued for variable periods (determined pragmatically depending on the initial burden and the extent of pathogenicity observed), and infection levels were examined at 7 day intervals. These methods have been reported more fully in the accounts of the individual experiments [2, 26, 27, 32, 33], and are summarised in Table 1.

### Statistical analyses and Bayesian models of gyrodactylid population growth

The Bayesian model for estimating gyrodactylid population growth rates was written and implemented using the WinBugs package [34, 35]. The null model for population growth in the absence of density- or time-dependent constraints is exponential (Additional file 1); the natural logarithm of parasite population size is linearly correlated with age of infection, and the slope of the relationship reflects population growth rate:

$$ \mathrm{r} = \ln\ \mathrm{N}\mathrm{t}-{\mathrm{N}}_0/\mathrm{t} $$

where r = the instantaneous growth rate of the parasite population, N_t_ is population size after time t and N_0_ is the starting population. The base model was developed from the Rats model of Lunn *et al*. [36], where the log_e_ parasite population size on time point i (mu_i_) was modelled as:

$$ {\mathrm{mu}}_{\mathrm{i}} = \mathrm{alpha} + \mathrm{beta}\left({\mathrm{x}}_{\mathrm{i}}\hbox{--} {\mathrm{x}}_{\mathrm{mean}}\right) $$

where alpha represented the initial parasite burden, beta the weekly growth rate, x_i_ the number of days at time point i, and x_mean_ the mean (mid) time point of the experimental series. Beta was centred on the midpoint of the time series to reduce posterior correlation between estimates of beta as recommended by Lunn *et al.* [36]. The observed parasite population at time point i, y_i_, was estimated from mu_i_ and a random variable tau, modelling observer error. Non-informative priors were used to initialise the simulation. After initialisation, 2 chains were run for 100 000 iterations, and the first 20 000 discarded as burn in. The chains were visually inspected to ensure convergence. Bayesian estimates of the population growth curve were used to estimate the regression curve for parasite populations on individual fish, and goodness of fit estimated using both a maximum likelihood sum of squares estimate (Σ(observed count – Bayesian estimated count)^2^) and a Bayesian estimate of the Deviance Information Criterion (DIC) implemented through the WinBugs package following a further 100 000 iterations (Table 2). Both the differences between sums of squares of predicted and observed population sizes (Δ_SSD_) were compared using likelihood ratio tests, and the differences between Bayesian DIC estimates (Δ_DIC_) were used to choose between models (Table 2). The exponential growth model was modified to take account of density-dependent and time-dependent growth effects. To estimate density-dependence, a term was included such that the exponent, beta, decreased at each time point of the growth curve by a quantity proportional to the size of the infection at the previous time point (Additional file 1). The time-dependent model was implemented by a time-dependent decreasing increment of beta such that beta declined throughout the infection (Additional file 1). These models allowed calculation of an individual parasite population growth rate for every fish at each time point in the infection. Full details of the models are given in the additional file.

**Table 2:** Fit of Bayesian models for exponential, time-dependent and density-dependent gyrodactylid population growth to experimental data-sets

Dataset	Fish stock and replicate	Exponential model	Time- dependent model	Density- dependent model	Reference
A	Loneelva single worm	SQ 1.2882	SQ 0.2542	SQ 3.6544	2
		DIC 29.61	DIC -20.70	DIC -8.667	
Bi	Batnfjordselva	SQ 72.825	SQ 0.345	SQ 1.665	Unpublished
		DIC 104.882	DIC – 4.452	DIC -3.380	
Bii	Lierelva	SQ 1.57	SQ 0.2135	Failed to converge	Unpublished
		DIC 15.464	DIC -38.464		
C	Imsa	SQ 2.4703	SQ 0.3912	SQ 2.4374	Unpublished
		DIC 25.839	DIC -80.125	DIC -36.162	
D	Altaelva	SQ 14.461	SQ 1.6153	Failed to converge	20
		DIC 157.55	DIC -135.64		
Ei	Akerselva	SQ 9.4279	SQ 3.2636	Failed to converge	Unpublished
		DIC 307.242	DIC 277.149		
Eii	Akerselva	SQ 1.453579	SQ 0.862444	Failed to converge	Unpublished
		DIC 169.796	DIC 80.931		
Eiii	Akerselva	SQ 9.767961	SQ 3.461779	Failed to converge	Unpublished
		DIC -157.044	DIC -154.38		
F	Conon	SQ 18.9395	SQ 5.2759	SQ 9.39509	19
		DIC 207.660	DIC 74.986	DIC 138.759	
G	Shin	SQ 68.0709	SQ 11.3739	Failed to converge	19
		DIC 394.026	DIC 139.259		
H	Numedalslågen	SQ 12.04599	SQ 6.240825	Failed to converge	10
		DIC 203.285	DIC 197.68		
I	Lierelva	SQ 28.8556	SQ 9.526	Failed to converge	19
		DIC 361.378	DIC 96.384		
J	Indalsälv	SQ 6.59083	SQ 4.396193	SQ 3.541148	21
		DIC 137.99	DIC 77.159	DIC 40.235	
K	Neva	SQ 80.1418	SQ 8.46197	Failed to converge	20
		DIC 198.842	DIC 120.456		
L	Neva single worm	SQ 17.6825	SQ 7.54459	Failed to converge	2
		DIC 86.573	DIC 57.064		
M	Neva	SQ 28.3822	SQ 8.2056	Failed to converge	Unpublished
		DIC 198.84	DIC 120.456		
N	Namsen	SQ 0.4047	SQ 0.1839	Failed to converge	Unpublished
		DIC 6.074	DIC 11.345		
O	Altaelva	SQ 4.0926	SQ 0.58324	Failed to converge	Unpublished
		DIC 123.98	DIC 11.626		

The Sum of Squares (SQ) and the Bayesian Deviance Information Criterion (DIC) are provided for each model

Having established the best model fitting the observed data, this model was used to calculate parasite population growth rates for every fish at each time point. Individual growth rates were compared using Analysis of Covariance (ANCOVA) implemented with analysis of variance (aov) performed in R 3.0 [37] using as a base model:

- Population growth rate~age of infection (Days) * stock * lon(initial population size)

The model was simplified with the step function in R, using comparisons of the Akaike Information Criterion (AIC) provided to establish minimum sufficient models.

## Results

**Fig. 1:**
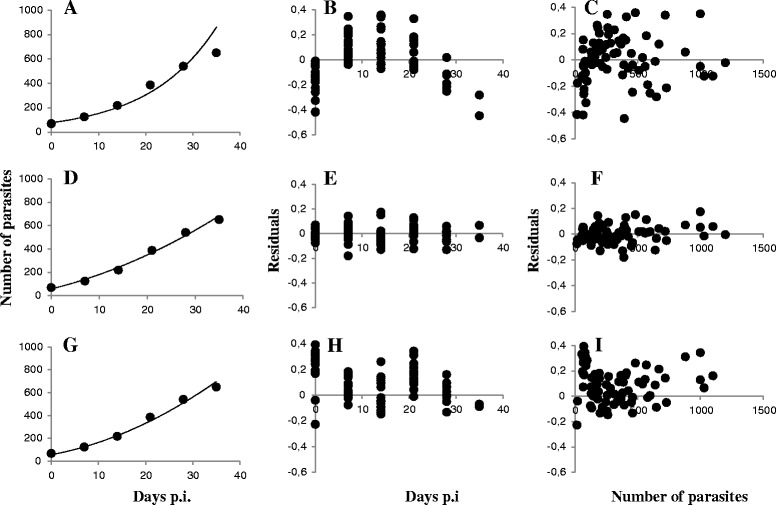
Fitting of Bayesian growth models to observed data for G. salaris infections on Atlantic salmon. **a-c**. A The Bayesian exponential growth model. **a** Sample fish infection data from Imsa dataset, fitted with the exponential model b Residuals from the Imsa dataset (18 fish) after fitting the exponential growth model, plotted against age of infection **c** the same residuals fitted against size of infection. **d-f**. The Bayesian time-dependent growth model. **d** sample fish infection data from Imsa dataset, fitted with the time-dependent model **e** Residuals from the Imsa dataset after fitting the time-dependent growth model, plotted against age of infection **f** the same residuals fitted against size of infection. **g-i**. The Bayesian density-dependent growth model. **g** Sample fish infection data from Imsa dataset, fitted with the density-dependent model. **h** Residuals from the Imsa dataset after fitting the density-dependent growth model, plotted against age of infection (**i**) the same residuals fitted against size of infection. Three very large residuals (>2) excluded from (**h**) and (**i**)

The base, exponential Bayesian model consistently overestimated parasite population size at later time points (Fig. 1a, b, c), and overall, all datasets were best fitted by the time-dependent growth model, which fitted data more closely, with smaller residuals showing a random distribution relative to age and size of infection, and smaller SSD and Bayesian DIC (Table 2, Fig. 1d, e, f, see Additional file 1). The density-dependent model fitted data better than the exponential model also (Fig. 1g, h, i), but never fitted as well as the time-dependent model (except for the Indalsälv dataset J). For several datasets the density-dependent model failed to converge and was unable to estimate parameters. The decline in parasite population growth rate throughout the infection therefore appears to be a general feature of all *G. salaris*-salmon interactions, but there is no evidence of density dependence beyond the trivial link between age of infection and parasite population size. This is especially the case for datasets, including Norwegian and Scottish fish stocks, where parasite populations began to decline again after a period of growth, which failed to converge with the density dependent model (Additional file 1).

Rates of parasite population growth rate were calculated for all twelve stocks using the time-dependent model of population growth, whereby the rate of growth (r) declines by a constant daily increment from the maximum achieved at the beginning of the experiment. Comparison of replicated stocks (Additional file 1), and of different stocks analysed under identical conditions in the same aquarium at the same time (datasets F, G and L at Vikan VESO; datasets B_i_ and B_ii_ at NHMO; datasets D and K, NHMO; datasets M and O, NHMO, see Table 1) give confidence that the initial (maximal) population growth rates of *G. salaris* are a valid measure of genetic differences between the fish stocks used, and make possible comparisons between stocks even when these were carried out at different times. The remaining variation observed was environmental, due to stochasticity in growth rates in small populations and to a significant effect of parasite inoculum on the initial population growth rate.

### Within stock effects

#### Stochasticity in small parasite populations

**Fig. 2:**
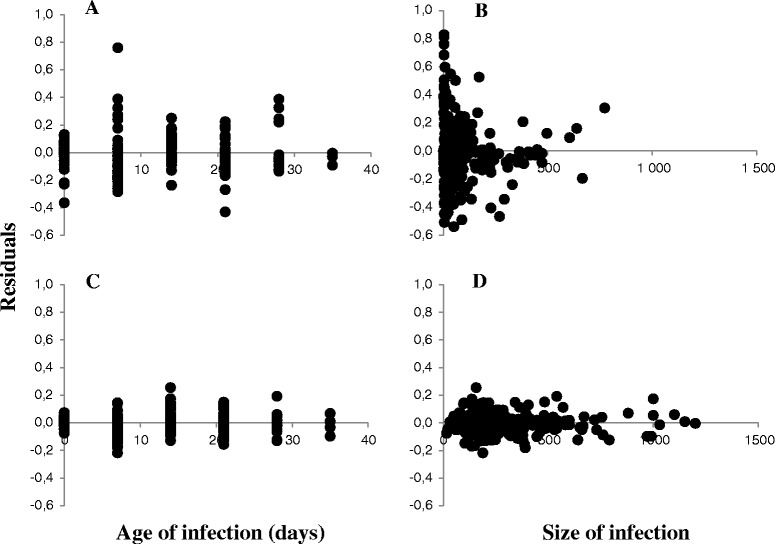
Stochastic variation in G. salaris infections on Atlantic salmon. **a-b**. Residuals from the time-dependent model for infections using large inocula (Akerselva 2, Loneelva, Imsa). **c-d**. Residuals from the time-dependent model for infections using small inocula (Numedalslågen, Akerselva 3, Loneelva single worm, Neva single worm). (**a**) and (**c**) residuals plotted against age of infection, (**b**) and (**d**) plotted against ln population size

In datasets A, E_iii,_ H, L (Table 1) in which infections started with small inocula of *G. salaris*, demographic stochasticity [29] contributed considerably to variation in initial parasite population growth rate. This can be seen from a comparison of residuals following fitting of the time-dependent model to stock datasets (A, E_iii,_ H, L) that started with small inocula ranging from 1 worm up to 44 (Fig. 2a, b), compared to those (B_i_, E_ii_, C) that started with large inocula ranging from 30–221 worms (Fig. 2c, d). The residuals are much larger, and show a much greater scatter at the beginning of the infection (when infections are small as predicted in [29]). Individual large residuals can, however, be observed for relatively long periods (Fig. 2a) because demographic stochasticity can maintain populations at a small size for considerable periods.

### Density-dependence at the beginning of the infection

**Fig. 3:**
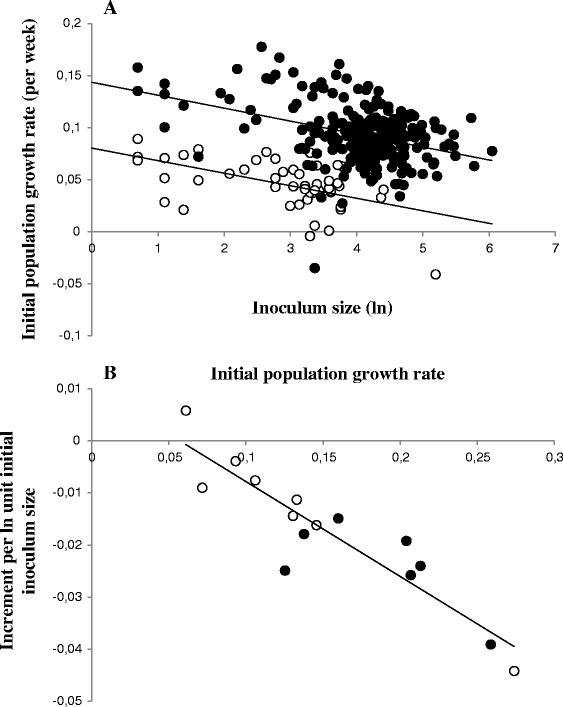
Effect of parasite density on initial population growth rate in G. salaris infections. **a** Relationship between initial population size and maximum (initial) growth rate for Neva (open circles), and Scottish and Norwegian stocks (filled circles). Pearson correlation coefficients for each group significant at the 0.05 level. **b** Relationship between mean maximum (initial) growth rate for each stock and the increment due to initial density (r^2^ = 0.83, P < 0.005). Filled circles, stocks in which the relationship was significant (P < 0.01) at the individual level, closed circles, stocks in which the relationship was not significant at individual level

Although there was no systematic evidence for density dependence during infections, parasite inoculum size did affect initial (maximum) rate of population growth in a density dependent manner. This was best seen in datasets in which the initial (maximum) rate of parasite population growth was high, for example in dataset B_i_ using Batnfjordselva (r^2^ = 0.5011, P < 0.01, n = 23) fishes. Datasets in which all fish were initially infected with the same or closely similar numbers of worms (datasets A ,Eiii, H, J, and L) were excluded from subsequent analysis, but when all remaining Norwegian/Scottish (217 fish) and Neva datasets (50 fish) were combined, a significant relationship between parasite inoculum size and initial population growth rate could be observed for both groups (Neva 50 fish, r^2^ = 0.361, P < 0.05; Norwegian/Scottish 217 fish, r^2^ = 0.2094, P < 0.05; Fig. 3a). This relationship was more obvious in the datasets with the highest initial rates of parasite population growth. When the mean decrease in growth rate due to parasite inoculum size for each dataset was plotted against mean initial growth rate, a highly significant relationship (r^2^ = 0.7546, P < 0.005) was noted, even when the relationship between parasite population growth rate and inoculum size within individual datasets was non-significant (Fig. 3b). These interactions between density and age of infection complicate analysis of stock effects in this system, because of the differences in starting density employed between different experiments.

### Between-stock variation

Using an Analysis of Covariance of all 18 datasets, fish stock identity accounted for 20 % of total variance, and age of infection for 40 %. All factors (fish stock, day (age) of infection and size of initial inoculum), and the interactions between them, were significant. The initial rates of parasite population growth are strongly affected by both fish stock (P < 2 × 10^-16^) and initial inoculum size (P < 2 × 10^-10^). The major coefficients are shown in Table 3.

**Table 3:** Factors influencing gyrodactylid population growth

Effect	Df	Sum of squares	Mean Square	F statistic	P	% variance accounted for
Age of infection	1	1.5875	1.5875	2571	2 × 10^-16^	22.8
Stock/replicate	17	3.555	0.20915	339	2 × 10^-16^	51.1
Initial inoculum	1	0.0251	0.0251	41	2 × 10^-10^	0.4
First order interactions						
Age of infection: stock	17	0.4306	0.02533	41	2 × 10^-16^	6
Age of infection: initial inoculum	1	0.0072	0.0072	11.7	0.00064	0.1
Stock: initial inoculum	14	0.0331	0.00236	3.8	1.8 × 10^-6^	0.05
Second order interactions						
Age of infection: stock: initial inoculum	14	0.0254	0.00181	2.9	0.00018	0.3
Residual (Unaccounted) variance	2095	1.2934	0.00062			18.6
Total		6.9578				100

Significant factors in ANCOVA model of gyrodactylid population growth rate ~ age of infection * stock * initial parasite inoculum

**Fig. 4:**
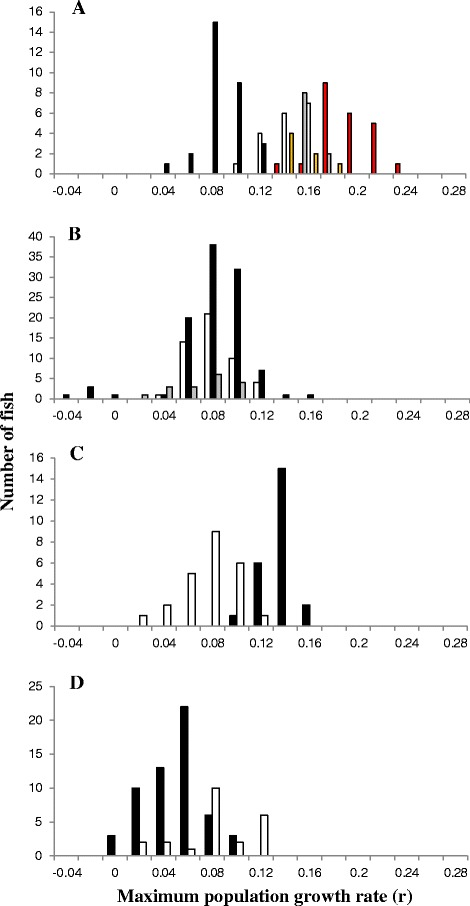
Frequency distribution of G. salaris population growth rates on different salmon stocks. **a** Northern and Western Norwegian stocks. Solid black bars, Altaelva; open bars Imsa; grey bars Loneelva; amber bars, Namsen; red bars, Batnfjordselva. **b** South-eastern Norwegian salmon stocks; solid bars, Akerselva stock, open bars Lierelva stock, grey bars Numedalslågen stock. **c** Scottish stocks; solid bars Shin stock, open bars Conon stock. **d** Baltic stocks; solid bars Neva stock, open bars Indalsälv stock

Overall, after correction for the effect of initial infection size, characteristic frequency distributions for initial parasite population growth rates on different fish stocks could be identified (Fig. 4a-d). The highest growth rates were observed in the western and northern Norwegian Imsa, Loneelva, Batn and Namsen River stocks (Fig. 4a), ranging between 0.09 and 0.24. The South-eastern Norwegian salmon stocks (Numedalslågen, Lierelva and Akerselva) supported a somewhat lower rate of parasite population growth ranging between -0.04 and 0.16 (Fig. 4b), as did the north Norwegian Alta stock (Fig. 4a). The two Scottish stocks supported a different spectra of parasite population growth rates; on the Conon stock (Fig. 4c) parasite populations grew at rates ranging from 0.02 to 0.12, similar to those seen on South-eastern Norwegian stocks, while the Shin stock supported a slightly higher parasite population growth rate, ranging between 0.09 and 0.16. Finally, the Baltic stocks (Indalsälv and Neva) supported relatively low growth rates ranging from 0 to 0.09 for Neva and 0.02 to 0.12 for Indalsälv (Fig. 4d).

For each host stock, a frequency distribution of parasite population growth rates were observed following fitting of the Bayesian time-dependent model. These frequency distributions contribute to a spectrum, with the fishes supporting the lowest parasite population growth rates observed in the Norwegian Akerselva stock, while the Baltic Indalsälv stock included fishes supporting growth rates overlapping those seen in Norwegian stocks. The frequency distribution of parasite population growth rates in the Akerselva salmon stock (Fig. 4b) has a wider range than that of other fish stocks, and shows some tendency to be polymodal.

**Fig. 5:**
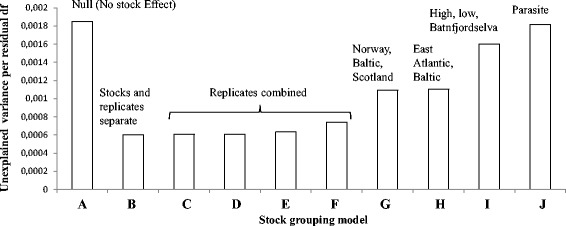
Effect of different grouping models on unexplained residual variance in G. salaris population growth rate. Models based on conventional groupings of Atlantic salmon according to race or geographical origin, or based on relative rate of population growth, fail to account for as much variance as the models in which all stocks are treated separately, or in which replicates (but not stocks) are grouped

Parasite population growth rates on particular host stocks were grouped to test hypotheses concerning the geographical origin of the fish. An ANCOVA in which the stock and replicate effect was ignored leaves 77 % of the total variance unexplained (Fig. 5a), whereas inclusion of all stocks and replicates leaves only 22 % of the total variance unexplained (P < 2 × 10^-16,^ ANCOVA, Fig. 5b). Stepwise combination of all possible replicates (Fig. 5c-f) results in models which also differ significantly (P < 2 × 10^-16^, ANCOVA) from the model (Fig. 5b) in which all replicates and stocks are treated separately, but the unexplained variance remains at only 26 %, further demonstrating that different replicates of the same host stock can be combined without losing predictive ability. Combination of stocks into groups predicted from phylogeography (Baltic, Norwegian and Scottish [Fig. 5g], or Baltic vs East Atlantic [Fig. 5h]), overall parasite population performance (high rate, low rate and Batnfjordselva [Fig. 5i]), or parasite strain (Lierelva Haplotype F, Figga Haplotype A or Batn Haplotype A [Fig. 5j]) in all cases failed to explain a minimum of 50 % of the total variance, giving them considerably less predictive power than the models in which all stocks were treated separately (Fig. 5b, with stocks and replicates treated separately, or 5 F, with only stocks treated separately).

## Discussion

This study represents the largest re-analysis of the growth dynamics of *Gyrodactylus salaris* on different Atlantic salmon stocks since the beginning of the epidemic in Norway in the mid-1970s. Prior to this study, the susceptibility of only six wild Norwegian [2, 21, 25–27, 32], two Baltic [2, 33] and two Scottish [26] salmon stocks had been published using the isolated, single fish, common garden methodology, a number of other Baltic and eastern Atlantic stocks have also been compared in complex multi-host arenas [38–40], and other stocks have been tested but the results remain unpublished. To place this in context, the epidemic in Norway has affected 44 rivers [1] out of 200+ rivers which support commercially and conservationally important salmon stocks, and so less than 10 % of Norwegian salmon stocks have been tested for their response to *G. salaris*; even with this re-analysis, the majority of stocks, in Norway and elsewhere, remain untested.

Remarkably, all but one dataset reanalysed here with Bayesian growth models showed strong support for time-limitation of parasite population growth rate, implying immunological response, and none, even amongst the Norwegian datasets, support prolonged exponential population growth by *G. salaris*. This is a similar conclusion to that reached by a smaller, non-Bayesian comparison of salmon stocks [21]. We can say unequivocally that unlimited population growth of *G. salaris* does not occur on Norwegian Atlantic salmon stocks, contrary to the widely held view of this interaction [20]. Instead, parasite population growth is limited by a complex interaction of declining population growth rates as the infection progresses, a density-dependent effect of parasite inoculum on initial (maximal) population growth rates, and stochastic effects limiting growth in small parasite populations [29]. The dependence of growth rate on infection age implies a common mechanism of parasite population regulation in Baltic and East Atlantic salmon stocks, contrary to the paradigm that ‘severe disease represents a lack of co-adaptation between the host and parasite’ [20]. In fact both Norwegian and Baltic salmon seem equally able to limit *G. salaris* population growth, as the rate of decline in the parasite growth rate is similar in fish stocks from both regions. The key difference is that initial parasite population growth rate is much higher on highly susceptible stocks. Nevertheless, even this statement must be interpreted with caution; to some extent, all salmon stocks show overlap in parasite population growth rates (Fig. 4), and the lowest initial rates were observed on individuals of the Norwegian Akerselva stock.

Previous interpretations of gyrodactylid population biology recognise exponential increase before an immune response eliminates parasites [15–19], and it is usually assumed that sterile immunity results [15], although this runs counter to observation, and has led various authors to postulate a ‘refractory period’, after which the fish once more becomes susceptible in the absence of further challenge [3, 9, 41]. The growth of *G. salaris* on Norwegian salmon stocks, is considered evidence of an impaired immune response [20]. The current work, however, suggests that *G. salaris* - Atlantic salmon interactions can be modelled conceptually in a different way. We envisage a maximum initial growth rate, set by host phenotype (including genetic and environmental components), which is reduced by a daily increment and will eventually become negative, leading to a decline in parasite population size. If the initial inoculum is large, initial parasite population growth rate may be limited in a density-dependent manner. On Neva fish, the initial growth rate is sufficiently low that population growth becomes negative after 10–30 days, and stochastic effects may dominate the interaction and maintain parasite populations at a very small size for considerable periods. Indeed, demographic stochasticity may play an important part in regulating gyrodactylid population growth when growth rates are very low; it is certainly likely to be important for example in maintaining prevalence and intensity of the Pålsbufjorden strain of *G. salaris* at very low levels on Arctic Charr [25, 32], and could represent a dominant form of population regulation in natural gyrodactylid populations. On Norwegian stocks supporting a higher initial growth rate, population growth remains positive for 40–50 days, and a decline in the infrapopulations is not often seen because fish death intervenes; on intermediate stocks such as the Norwegian Lierelva or the Scottish Conon stocks, host survival is such that parasite population growth rate does become negative, and the parasite population begins to decline, a fact noted in the original study by Bakke and Mackenzie [26].

In natural populations, the response to gyrodactylids is complex; most fish remain infected even though, given their age and history of exposure, they should have responded to infection [8, 14, 42, 43]. This is actually the case for *G. salaris*, at least in Southern Norway, where in the Lierelva River most infected fish carried declining parasite populations by midsummer [44, 45], in line with predictions of a 50 day threshold before parasite population growth rates become negative in this stock. Non-sterile immunity is characteristic of gyrodactylids, and a model of the immune response, which limits parasite population growth, but does not necessarily eliminate the *Gyrodactylus* population entirely, is more realistic than the rather crude search for ‘resistance genes’ which has been undertaken to date [46, 47]. The reanalyses presented here suggest that immune activation after a lag phase does not take place, and that the response is apparent from the earliest stages of the infection. This strongly argues against a specific response in favour of expression of a non-specific effector, capable of pattern recognition and response, similar perhaps (but not exclusive to) to alternate pathway complement activation against gyrodactylids [48, 49]. When parasite challenge is low, expression of the response declines and the fish become phenotypically more susceptible; when the parasite challenge is high, expression increases, reducing susceptibility, but at no point does the fish become entirely refractory, and neither does it lose all resistance if challenge ends. This owes much more to the model of the gyrodactylid host interaction of Lester and Adams [3] than it does to the compartmentalisation of fish as naïve, infected or refractory [41], based on immune processes in tetrapods, or to the unrealistic expectations of sterile immunity seen in the veterinary and fish health literature [15, 17–19, 47].

In all stocks tested, a spectrum of initial parasite population growth rates was observed. Trials using the same fish stock conducted several years apart showed sufficient repeatability, while results with different stocks at the same time in the same facility were sufficiently different to consider that observed stock differences were not artefacts. This also strongly argues the case for a host genetic component influencing parasite population growth characteristics, and allows a quantitative genetic approach to be adopted. In most cases, initial parasite population growth rates follow a unimodal distribution (Fig. 4), suggesting complex polygenic control of susceptibility, but in the Akerselva stock, and to some degree in the Indalsälv stock, initial growth rates were polymodal and suggestive of an additive genetic basis for parasite population growth, similar to that noted by Madhavi and Anderson [9] for guppies infected with *G. turnbulli*. The Akerselva dataset is most striking; this included two fish which sustained parasite population growth rates similar to those seen in the most susceptible northern and western Norwegian stocks, but it also included fishes supporting the lowest growth rates ever noted, lower even than those seen in Baltic stocks. It is not clear why these extreme individuals were not observed in other fish stocks, and it may be relevant that the Akerselva study was composed of fry and alevins rather than parr, or that the Akerselva has a history of introduction, including Baltic Neva fishes [50]. Equally however, the Akerselva study represented the largest group of fish analysed in the present work (100 fish), and it may simply require samples of this size to reveal the full range of phenotypes present in a fish stock.

The original host phylogeographic hypothesis, based on protein allozyme electrophoresis [51] and RFLP analysis of mitochondrial DNA [52], suggested that the Atlantic salmon is broadly distributed into three major races (West Atlantic, East Atlantic and Baltic), and the first experimental study of *G. salaris* population growth on individually isolated fish included the Norwegian Loneelva stock and a Baltic salmon stock from the Russian river Neva [2]. The ability of the Neva stock to control their infections supported the paradigm of a resistant Baltic salmon race, which have long prior evolutionary experience of the parasite, and of a susceptible, previously unexposed East Atlantic race (see [1, 21]). We found no evidence supporting the partitioning of resistance / susceptibility between salmon stocks according to phylogeographic hypotheses. Differences in *G. salaris* population growth rate cannot be predicted based on a partitioning of stocks between East Atlantic and Baltic salmon races (the Bakke *et al*. [2] hypothesis), or between the three geographical areas considered in this work, Norway, Scotland and the Baltic. The diversity of *G. salaris* growth rates on different stocks most strongly support the view central to conservation of the species, that the Atlantic salmon comprises multiple, genetically differentiated and reproductively isolated, populations within, as well as between, major river systems [51, 53–55]. At the same time, the results reject local adaptation in response to parasite selective pressure; the heterogeneity of parasite growth rates on Norwegian and Scottish salmon stocks, and especially the relatively low growth rates supported by South-eastern Norwegian stocks, which according to the paradigm have no prior experience of the parasite, argues strongly against local adaptation specifically to *G. salaris*. It could be that local adaptation takes place in response to locally distributed disease syndromes, and that for example the Scottish Conon stock supports low growth of *G. salaris* because of prior evolutionary experience of other pathogens. In fact, most Atlantic salmon stocks have evolutionary experience of gyrodactylids; *Gyrodactyloides bychowskii* Albova, 1948 is present in the marine phase [56], infections with *Gyrodactylus derjavinoides* Malmberg *et al*., 2007 or *G. teuchis* Cunningham *et al*., 2001 are frequent in rivers containing brown trout [57, 58] and when surveyed, 50 % of Scottish salmon populations were infected with either *G. derjavinoides* or *G. caledoniensis* Shinn *et al*., 1995 [59]. The poor performance on Norwegian South-eastern stocks may reflect past experience of *G. salaris*, as this region is relatively close to the current northern limit of the natural range, or genetic exchange with salmon stocks from rivers historically infected with *G. salaris* along the Swedish west coast. This may therefore have conferred some degree of resistance into salmon from the South-eastern Norwegian rivers.

## Conclusions

The current Bayesian analysis shows that the control of *G. salaris* infrapopulation growth is complex, involving stochasticity, time-dependence, and density-dependence limiting population growth on fish receiving the largest inocula. These features are present even in fish stocks with no recent evolutionary experience of the parasite, suggesting that they are common to all *S. salar* stocks. A spectrum of phenotypes is present, and is shaped by natural selection, either in response to *Gyrodactylus* infection, or to proxy infections. However, we find no evidence to support the ‘Baltic resistant, Norwegian susceptible’ paradigm which has come to dominate this topic, and rather note that some South-eastern Norwegian salmon stocks, with no evolutionary experience of the parasite, are at least as resistant as some Baltic salmon stocks which are thought to have co-evolved with *G. salaris*.

## Additional file

Additional file 1:
**WinBUGS script and the justification for the Bayesian models.** Programming script for the WinBUGS Bayesian population growth models utilised in the study (exponential, density-dependent, time-dependent).
