# Supplementary material for: Population regulation in Gyrodactylus salaris – Atlantic salmon (Salmo salar L.) interactions: testing the paradigm
Source: Parasit Vectors. 2015 Jul 25;8:392. doi: 10.1186/s13071-015-0981-4 (PMC4513975; doi:10.1186/s13071-015-0981-4)
Supplement: Additional file 1: — WinBUGS script and the justification for the Bayesian models. Programming script for the WinBUGS Bayesian population growth models utilised in the study (exponential, density-dependent, time-dependent). [file 13071_2015_981_MOESM1_ESM.pdf]

Additional file 1 – validation of the Bayesian approach and model fitting

The Bayesian growth model – fit to observed data

For datasets B_i, D, E_i, E_{iii}, G, I, K, L and M, the exponential model was rejected unequivocally in favour of the time dependent model on the basis of both Δ_{SSD} (Likelihood Ratio Tests, P>0.01), and Δ_{DIC}, which following [24] exceeded -10. For all remaining datasets except two (datasets H and N), $\Delta_{DIC} < 10$ and the exponential model was rejected in favour of the time-dependent model although Δ_{SSD} was not significant with the Likelihood Ratio Test. In only two datasets (H, Numedalslågen, and N, Namsen) was the difference in Δ_{DIC} between exponential and time dependent models less than 10. For dataset H, ΔDIC was 6; following [24] this represents a substantial likelihood ratio difference, and as ΔSSD was close to significance (LRT value =5.876; critical region for P>0.01 is 6.635) we reject the exponential model for this dataset also. The only dataset for which it was not possible to reject the exponential model was the Namsen (dataset N) group. This was the smallest cohort of fish (7 fish), followed for the shortest period of time (21 days) and this may simply reflect an inadequate sample size to detect any time-dependent effects. Overall then, we favour the time dependent model over the exponential growth model for all datasets.

The density-dependent model also gave a significantly better fit than the exponential model as evaluated by both Δ_{SSD} and Δ_{DIC}, but only for the Indalsälv dataset (dataset J) was it judged better than the time-dependent model ($\Delta_{DIC\,=}$ -37 relative to the time-dependent model, not supported by Δ_{SSD}). For all other datasets it fitted less adequately than the time-dependent model, with larger residuals. For datasets in which the parasite population began to decline after a period of initial growth (datasets E_i,E_{ii},E_{iii},F, G, I, K,L,M,), the density-dependent model failed to converge on a satisfactory solution. Plotting of residuals after fitting of the time dependent model against parasite density also failed to reveal a trend (data not shown), suggesting that a density-dependent effect was not masked by the time-dependent limitation

in parasite population growth rate. Given the relative performance of these three models, the time-dependent model was therefore chosen as that with the greatest explanatory power, and was applied to all datasets.

Validation of the experimental methodology

The datasets available provided an opportunity to compare *G. salaris* population growth on the same fish stock at different times, allowing the repeatability of the experimental approach to be evaluated. Two replicates with Altaelva stock were carried out (dataset D, performed in 1994 and O, dating from 1995), both using the Lierelva strain of parasites. *G. salaris* showed a significantly higher maximum rate of population growth in the second experiment ($P = 4 \times 10^{-6}$, ANCOVA), accounting for 35% of the variance across the two replicates (Additional file, Figure 1A). However, using an ANCOVA of the form:

population growth rate ~ day of infection * replicate * ln(population size at the start of the infection)

it was clear that the difference between replicates was due to the difference in the starting infections; the difference between the replicates was not significant when interactions between replicate and starting infection size were taken into account.

The two replicates with Lierelva stock (dataset Bii, carried out in 1993 and I, 1992) used different haplotype A parasite strains. Again, *G. salaris* performed differently, with dataset Bii showing a smaller decline in growth rate with infection age than dataset I (Additional file, Fig. 1B). However, the difference in initial growth rates between the datasets was insignificant ($P = 0.408$, ANCOVA).

The three datasets using Neva fish (datasets K, 1994; dataset L, 1989; dataset M, 1995) included the original data (dataset L) used by Bakke et al. [2] to infer resistance in this stock of fish. In this case, the differences between replicates could not be simply explained in terms of the explanatory variables (age of infection or initial burden), and a total of 40% of the variance remained unexplained (Additional file, Figure 2A).

The final set of replicate datasets (Ei, Eii, Eiii) used alevins/fry from the Akerselva River, performed during summer 1998 (Additional file, Figure 2B). The replicates show different outcomes, which could not be accounted for by differences in initial parasite inoculum. In this case, replicate accounted for only 20% of the total variance, and the significant differences between the three replicates were due to the presence in two replicates of a small number of fish supporting low parasite population growth rates (Additional file, Figure 2B). Consideration of growth rates at the beginning of the experiment revealed no significant difference between replicates.

Overall then, effects of replicate on initial parasite population growth rate, after accounting for differences in parasite starting density, were not significant, and comparison of initial parasite population growth rates within and between replicates, after exclusion of effects due to starting density, provides an effective tool for analysis of stock differences in susceptibility to *G. salaris*.

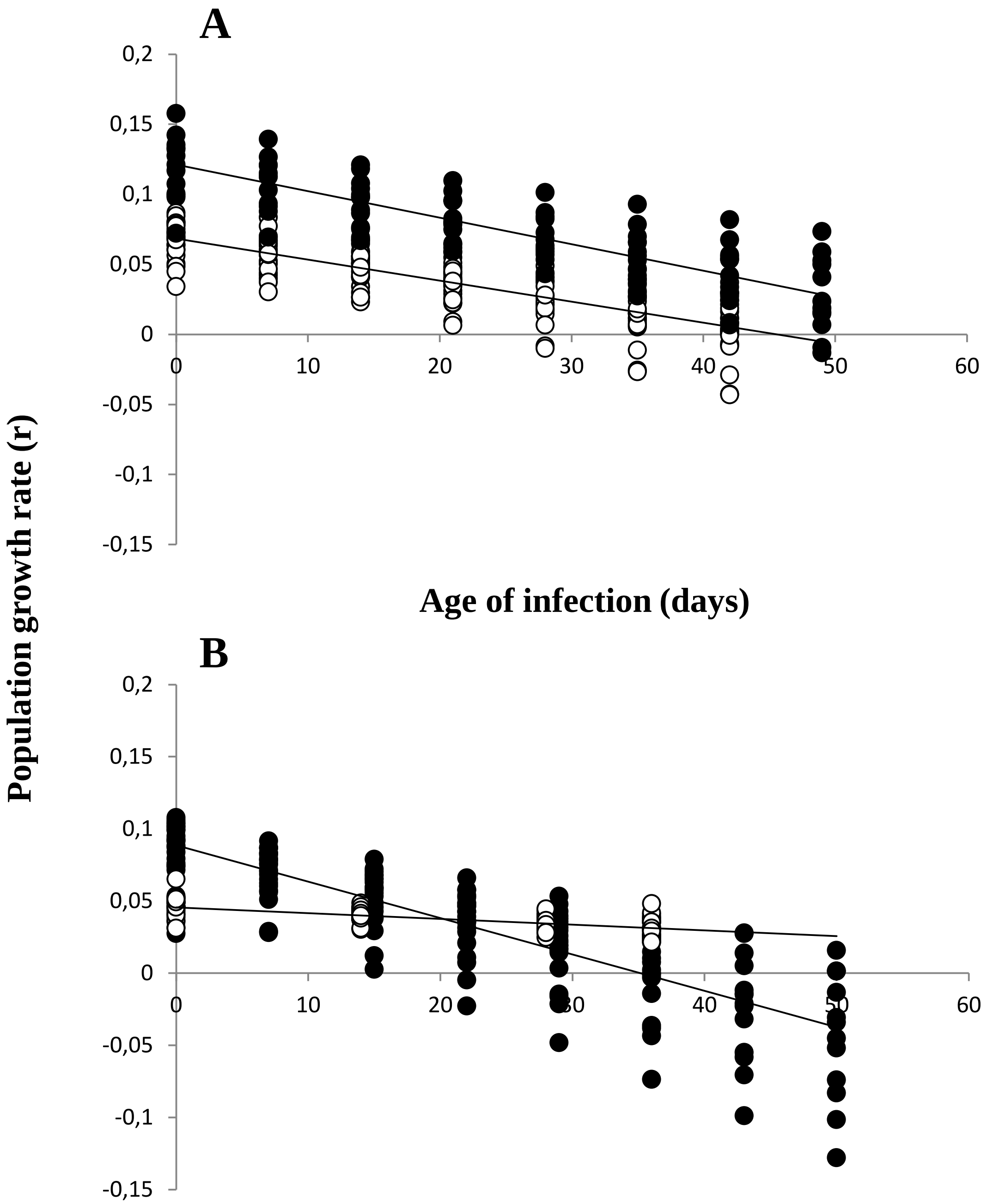
A
B
Population growth rate (r)
Age of infection (days)
0,2
0,15
0,1
0,05
0
-0,05
-0,1
-0,15
0
10
20
30
40
50
60

Additional file, Figure 1 - Performance of *G. salaris* on Altaelva and Lierelva salmon stocks

(A) Comparison of two replicates carried out at different times of *G. salaris* growing on the Altaelva stock of Atlantic salmon. Solid circles, 12 fish used as negative controls in an unpublished challenge experiment; open circles, 24 fish used as controls in the salmon x brown trout hybrid experiments (respectively, Altaelva x Fossbekk River stock, South-western Norway) described by Bakke et al. [20]. (B) Comparison of two replicates carried out using the Lierelva stock of Atlantic salmon [2] infected with Figga (haplotype A, solid circles) or Batnfjordselva (haplotype A, open circles) parasites. In both stocks performance of the parasite in the two replicates is significantly different, but the difference in initial (maximum) population growth rate can be attributed entirely to differences in the size of the founding populations.

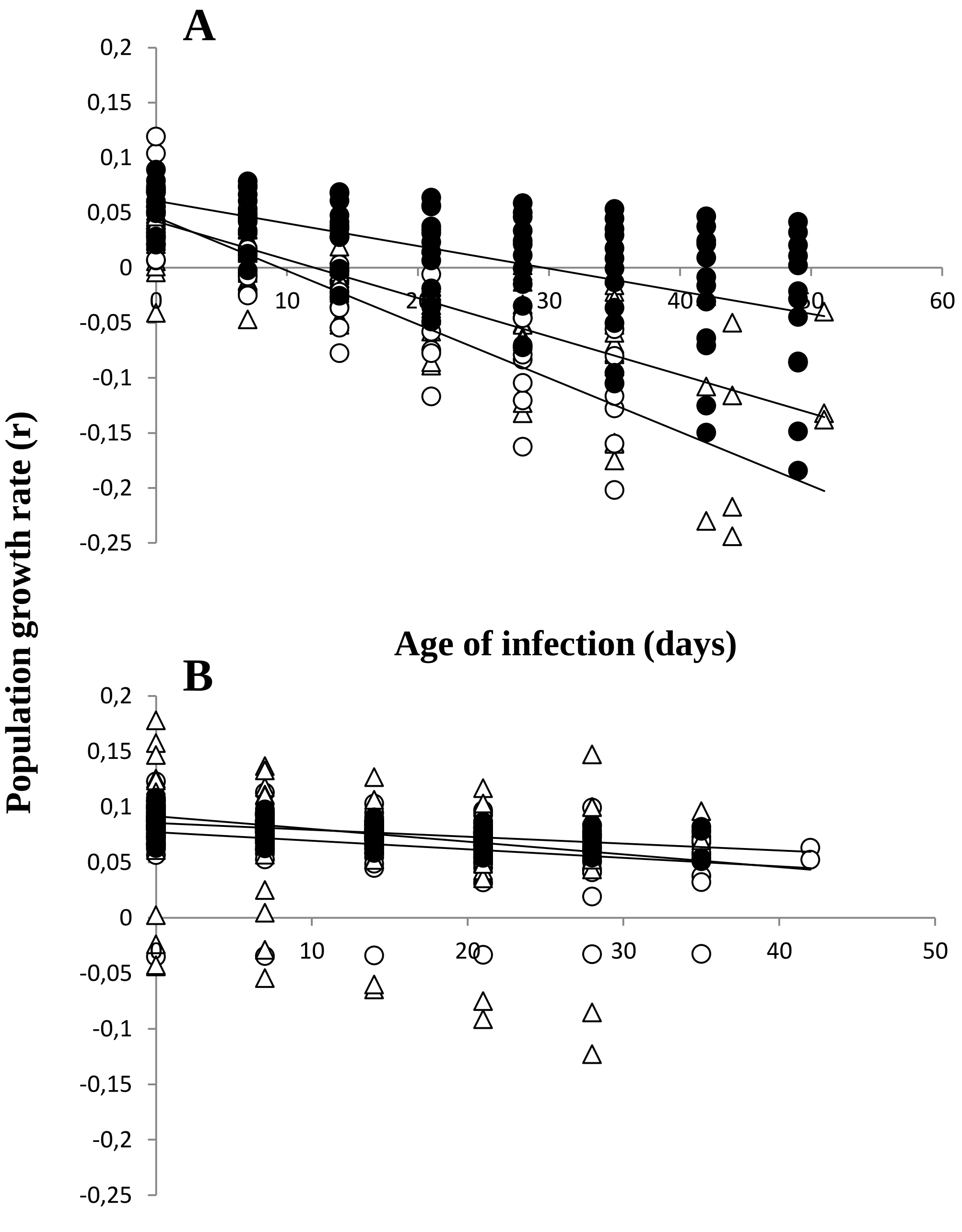
A
B
Population growth rate (r)
Age of infection (days)
0,2
0,15
0,1
0,05
0
-0,05
-0,1
-0,15
-0,2
-0,25
0
10
20
30
40
50
60

Additional file, Figure 2 - Performance of *G. salaris* on Neva and Akerselva salmon stocks

(A) On a Neva stock of salmon. Solid circles dataset M, open circles dataset L (single worm infections) and open triangles dataset K; (B) On the Akerselva stock of salmon. Solid circles dataset Ei, open circles dataset Eii, open triangles dataset Eiii.

1. WinBUGS script for the Bayesian model of exponential gyrodactylid population growth.

```
model
	{
		for( i in 1 : N ) {
			for( j in 1 : T ) {
				Y[i , j] ~ dnorm(mu[i , j],tau.c)
				mu[i , j] <- alpha[i] + beta[i] * (x[j] - xbar)
			}
			alpha[i] ~ dnorm(alpha.c,alpha.tau)
			beta[i] ~ dnorm(beta.c,beta.tau)
		}
		tau.c ~ dgamma(0.001,0.001)
		sigma <- 1 / sqrt(tau.c)
		alpha.c ~ dnorm(0.0,1.0E-6)
		alpha.tau ~ dgamma(0.001,0.001)
		beta.c ~ dnorm(0.0,1.0E-6)
		beta.tau ~ dgamma(0.001,0.001)
		alpha0 <- alpha.c - xbar * beta.c
	}
```

2. WinBUGS script for the Bayesian model of density-dependent gyrodactylid population growth.

```
model
	{
		for( i in 1 : N ) {
		oldmu[i,1]<-0
			for( j in 1 : T ) {

				Y[i , j] ~ dnorm(mu[i , j],tau.c)
			   omega[i , j] <- beta[i] - density[i]*oldmu[i,j]
				mu[i , j] <- alpha[i] + omega[i,j] * (x[j] - xbar)
				oldmu[i, j +1]<-mu[i, j]
							}
			alpha[i] ~ dnorm(alpha.c,alpha.tau)
			beta[i] ~ dnorm(beta.c,beta.tau)
			density[i] ~ dnorm(density.c,density.tau)
						}
		tau.c ~ dgamma(0.001,0.001)
		sigma <- 1 / sqrt(tau.c)
		alpha.c ~ dnorm(0.0,1.0E-6)
		alpha.tau ~ dgamma(0.001,0.001)
		beta.c ~ dnorm(0.0,1.0E-6)
		beta.tau ~ dgamma(0.001,0.001)
		density.c ~ dnorm(0.0,1.0E-6)
		density.tau ~ dgamma(0.001,0.001)
		alpha0 <- alpha.c - xbar * beta.c
	}
```

3. WinBUGS script for the Bayesian model of time-dependent gyrodactylid population growth.

```
model
	{
		for( i in 1 : N ) {
			for( j in 1 : T ) {

				Y[i , j] ~ dnorm(mu[i , j],tau.c)
			   omega[i , j] <- beta[i] -(time[i]*(x[j] - xbar))
				mu[i , j] <- alpha[i] + omega[i,j] * (x[j] - xbar)
							}
			alpha[i] ~ dnorm(alpha.c,alpha.tau)
			beta[i] ~ dnorm(beta.c,beta.tau)
			time[i] ~ dnorm(time.c,time.tau)
						}
		tau.c ~ dgamma(0.001,0.001)
		sigma <- 1 / sqrt(tau.c)
		alpha.c ~ dnorm(0.0,1.0E-6)
		alpha.tau ~ dgamma(0.001,0.001)
		beta.c ~ dnorm(0.0,1.0E-6)
		beta.tau ~ dgamma(0.001,0.001)
		time.c ~ dnorm(0.0,1.0E-6)
		time.tau ~ dgamma(0.001,0.001)
		alpha0 <- alpha.c - xbar * beta.c
	}
```
